# Local Sustained Delivery of Temozolomide via an Injectable Poly(Anhydride-Ester) Depot for Glioblastoma Therapy

**DOI:** 10.3390/pharmaceutics18080963

**Published:** 2026-08-05

**Authors:** Hasan Slika, Christine Warwar Damouny, Aanya Shahani, Harshal A. Shah, William ElNemer, Esteban Velarde, Christopher Peters, Omar Selim, David Lee, Toriyn Dotson, Charles G. Eberhart, Peter Siman, Henry Brem, Abraham Domb, Betty Tyler

**Affiliations:** 1 Hunterian Neurosurgical Laboratory, Department of Neurosurgery, School of Medicine, The Johns Hopkins University, Baltimore, MD 21231, USA; hslika1@jh.edu (H.S.);; 2 Intragel Therapeutics Ltd., Wadi El Haj, Nazareth 17111, Israel; christine@intra-gel.com (C.W.D.);; 3 Department of Radiation Oncology and Molecular Sciences, School of Medicine, The Johns Hopkins University, Baltimore, MD 21231, USA; 4 Department of Pathology, School of Medicine, The Johns Hopkins University, Baltimore, MD 21231, USA; 5 Departments of Ophthalmology, Oncology, and Biomedical Engineering, The Johns Hopkins University, Baltimore, MD 21231, USA; 6 Department of Medicinal Chemistry and Natural Products, School of Pharmacy, The Hebrew University, Jerusalem 9112001, Israel; 7 Faculty of Health, Jerusalem College of Technology, Jerusalem 9372115, Israel

**Keywords:** local delivery, biodegradable polymer, glioblastoma, translational neuro-oncology, adjuvant therapy

## Abstract

**Background/Objectives:** Glioblastoma (GBM) remains one of the most aggressive primary brain malignancies, with limited therapeutic progress over the past two decades. Systemic administration of temozolomide (TMZ) is a pillar of clinical management but is constrained by poor brain penetration, short half-life, and systemic toxicity. Localized drug delivery systems represent a compelling approach to address these limitations. We report the development and evaluation of an injectable poly(sebacic acid–ricinoleic acid) poly(anhydride-ester) (pSARA) gel for sustained intratumoral delivery of TMZ. **Methods:** The pSARA gel was synthesized using a one-pot melt polycondensation technique, and its in vitro release dynamics were assessed using spectrophotometry. In vivo efficacy of the TMZ-loaded pSARA gel was evaluated as a monotherapy and as an adjuvant to radiation or surgical resection using an orthotopic 9L gliosarcoma rat model. **Results:** The formulation exhibits shear-thinning behavior, enabling syringe-based administration, and undergoes surface erosion in aqueous environments to achieve controlled drug release. In vivo, the TMZ-loaded pSARA significantly prolonged survival compared to controls and outperformed paclitaxel-loaded formulations. Furthermore, combination therapy with radiation or surgical resection demonstrated combined survival benefits, including long-term survivors. **Conclusions:** These findings highlight the translational potential of pSARA-based local delivery systems as an adjunct or alternative to systemic chemotherapy in GBM treatment.

## 1. Introduction

Glioblastoma (GBM) is the most prevalent and aggressive primary malignant tumor of the central nervous system, notable for its extensive infiltration, high proliferative capacity, and inevitable recurrence [1]. Despite multimodal treatment approaches—including maximal surgical resection, radiotherapy, and chemotherapy—the median survival is approximately 14–18 months, and the 5-year survival rate remains below 10% [1,2]. Since the introduction of concomitant radiotherapy and temozolomide (TMZ) in 2005, no major paradigm shift has significantly improved patient outcomes [3].

A central challenge in GBM therapy is the presence of the blood–brain barrier (BBB), which severely restricts the penetration of systemically administered therapeutics into the tumor microenvironment [4]. TMZ, a small lipophilic alkylating agent, partially overcomes this limitation; however, its clinical efficacy is still constrained by suboptimal intratumoral concentrations, rapid systemic clearance (half-life ~1.8 h), and dose-limiting toxicities such as myelosuppression and hepatotoxicity [5,6]. These limitations necessitate repeated high-dose administration, further exacerbating systemic side effects without guaranteeing adequate tumor exposure. Local delivery of TMZ using polymeric wafers for treating brain tumors in a rat animal model has been demonstrated [7].

Local drug delivery strategies have emerged as a compelling alternative to bypass the BBB and achieve high intratumoral drug concentrations while minimizing systemic toxicity [8]. The clinical approval of carmustine-loaded wafers (Gliadel^®^) demonstrated the feasibility of this approach [9]. However, rigid wafer-based systems suffer from several drawbacks, including limited conformity to the resection cavity, uneven drug distribution, and burst release kinetics due to high surface-area-to-volume ratios [10]. These limitations have prompted the development of next-generation delivery systems with improved adaptability and controlled release characteristics.

Injectable polymeric depots, particularly gel-based systems, offer several advantages over solid implants. These include conformal filling of irregular surgical cavities, minimally invasive administration, and tunable physicochemical properties [11]. Among these, fatty acid-based poly(anhydride-ester) systems have gained considerable attention due to their predictable surface erosion behavior, biocompatibility, and ability to achieve near zero-order drug release [12,13]. Importantly, surface-eroding polymers maintain structural integrity during degradation, enabling controlled and sustained release profiles that are highly desirable for local chemotherapy.

The poly(sebacic acid–ricinoleic acid) (pSARA) platform represents a unique class of injectable poly(anhydride-ester) materials that combine hydrophobicity, biodegradability, and shear-thinning rheological behavior [14]. Upon exposure to aqueous environments, pSARA undergoes hydrolytic degradation primarily at the surface, allowing controlled release of encapsulated therapeutics [15]. This platform has demonstrated promising results in multiple preclinical disease models, including cancer and inflammatory disorders, and has recently advanced into clinical evaluation [16].

In this study, we investigate the use of a TMZ-loaded pSARA injectable depot for localized treatment of GBM. We hypothesize that sustained intratumoral delivery of TMZ via a surface-eroding polymer matrix will enhance therapeutic efficacy. Using an orthotopic 9L gliosarcoma model, we evaluate the efficacy of TMZ-loaded pSARA as a monotherapy and in combination with clinically relevant modalities, including radiation therapy and surgical resection. Additionally, we compare its performance with a paclitaxel (PTX)-loaded formulation to assess drug-dependent release and therapeutic outcomes.

## 2. Materials and Methods

### 2.1. Gel Preparation

The synthesis of the gel was conducted using a one-pot melt polycondensation technique as described in previous publications [12,17]. Initially, ricinoleic acid (RA) and sebacic acid (SA) underwent esterification at 160 °C under vacuum to generate RA-SA oligomeric chains. These intermediates were subsequently acetylated using acetic anhydride at 160 °C. Following the removal of acetic acid and residual anhydride via vacuum distillation, the reaction proceeded at 160 °C to finalize the polymerization. The resulting polymer, formed at a 30:70 SA:RA molar ratio, is a lipophilic, gel-like material at room temperature that exhibits increased viscosity when exposed to aqueous environments [18].

### 2.2. Drug-Loaded Gels

Polymer formulations loaded with TMZ or PTX were prepared. The drug and the gel were mixed at room temperature using a mortar and pestle until a uniform paste was obtained. The resulting formulation was packed into 1 mL Luer-lock syringes (BD Hypak, Becton, Dickinson and Company, Franklin Lakes, NJ, USA) and stored at −20 °C until use. The filled syringes were sterilized by gamma irradiation (25–45 kGy) under dry ice to minimize thermal degradation and stored at −20 °C.

### 2.3. In Vitro TMZ Drug Release

Due to TMZ instability in PBS buffer medium, in vitro release studies were conducted in acetate buffer (pH 5.0) at 37 °C for up to 10 days, following previously reported methods [19]. Briefly, 100 mg of the formulation (TMZ loading of 10% or 30% *w*/*w*) was immersed in 50 mL of release medium and incubated under controlled conditions. At predetermined time points, aliquots of the medium were withdrawn, and the concentration of released TMZ was determined by measuring absorbance at 328 nm using a UV/Vis spectrophotometer (Thermo Scientific GENESYS 50, Thermo Fisher Scientific, Waltham, MA, USA). All experiments were performed in triplicate. Sink conditions were maintained throughout the study as TMZ concentrations remained well below its solubility in acetate buffer.

### 2.4. In Vitro PTX Drug Release

In vitro release of PTX from the polymer was evaluated in phosphate-buffered saline (PBS, pH 7.4) at 37 °C. A 100 mg depot formulation was fully immersed in 50 mL of PBS and incubated at 37 °C. At defined intervals, the entire PBS medium was collected and replaced with fresh PBS to maintain sink conditions. The PTX released into the aqueous medium was quantified by measuring its absorbance at 230 nm (UV-Vis spectrophotometry). All measurements were done in triplicate, and results were reported as the mean ± standard deviation.

### 2.5. In Vivo Tumor Model Generation

All animal-related studies and procedures were performed in compliance with the guidelines of the Johns Hopkins University Animal Care and Use Committee. Female Fischer 344 rats, weighing 125–150 g, were acquired from Charles River Laboratories and used for all in vivo experiments. The rats were housed in the animal vivarium with regulated light-dark cycles and free access to water and food.

For the generation of the model, the 9L glioblastoma tumor model was used. These tumors are maintained and passaged in the flanks of donor F344 rats every 2–3 weeks. As needed for intracranial implantation, the donor rats are euthanized, and their tumors are excised using sterile procedure. The tumors are cut into 2 mm^3^ pieces and placed in sterile normal saline on ice.

For the tumor implantation procedure, rats were anesthetized via an intraperitoneal injection of a mixture of ketamine (75 mg/kg), xylazine (7.5 mg/kg), and ethanol (14.2%) in 0.9% sterile saline. For additional analgesia, the rats received a subcutaneous injection of long-acting buprenorphine (0.1 mg/kg). After confirmation of proper anesthesia, the scalp was shaved and prepped with ethanol and Prepodyne. A midline incision was made, bregma was located, and a burr hole (2 mm in diameter) was drilled with its center 2.5 mm left and 2.5 mm posterior to bregma using a Foredom K.1070 Micromotor drill (Foredom, Bethel, CT, USA). Under the guidance of Zeiss Opmi surgical microscope (Zeiss, Oberkochen, Germany), a small area of the cortex is resected, and one of the harvested 9L tumor pieces is implanted within the parenchyma. Finally, the incision is closed using sterile surgical clips.

### 2.6. Comparing the Efficacy of Temozolomide Versus Paclitaxel-Loaded Gels

Five days following tumor implantation, 16 rats were randomized into 3 groups: (1) Control (*n* = 5), (2) TMZ-loaded gel (*n* = 5) and (3) PTX-loaded gel (*n* = 6). For groups 2 and 3, the rats were anesthetized, their staples removed and incisions reopened. The animal was mounted on a stereotactic frame, and the same burr hole created initially was used to insert a 19G needle fitted onto a 1 mL syringe loaded with 30 µL of the gel. The tip of the needle was lowered 2.5 mm below the surface of the brain, and the gel was injected slowly over 1 min. Subsequently, the rats were unmounted from the frame, and their incisions re-stapled.

### 2.7. Combination with Radiation Therapy

To test the efficacy of combining the TMZ-loaded gel with radiation, 22 rats were randomized into 3 groups: (1) Control (*n* = 7), (2) Radiation (*n* = 8) and (3) Radiation + TMZ-loaded gel (*n* = 7). For the radiation therapy, a dose of 10 Gy was given to the rats on day 6 after tumor implantation using an XSTRAHL Small Animal Radiation Research Platform (SARRP) [20,21,22]. In specific, a 10 × 10 mm X-ray beam was utilized, and it was targeted 3 mm inferior to the drilled hole in the skull based on a computed tomography scan. The rats randomized to the radiation + TMZ-loaded gel group had received the intratumoral injection of the gel as described previously on day 4 following tumor implantation.

### 2.8. Combination with Tumor Resection

To investigate the utility of the TMZ-loaded gel as an adjuvant therapy to surgical resection, 16 rats were randomized into the following groups: (1) Resection + Empty gel (*n* = 8) and (2) Resection + TMZ-loaded gel (*n* = 8). The surgical resection was performed on day 5 following tumor implantation. Briefly, the rats were anesthetized as described previously, with their wounds prepped with ethanol and Prepodyne and reopened. Under the guidance of Zeiss Opmi surgical microscope, the tumors were resected using a Pasteur pipette connected to a vacuum suction pump. The TMZ-loaded gel or empty gel was injected into the resection cavity after achieving hemostasis. The wounds were subsequently closed, and the rats were monitored until they emerged from anesthesia.

### 2.9. Histological Analysis

Upon euthanasia, brains of the rats were harvested and fixed in formalin and subsequently embedded in paraffin. The paraffin blocks were sectioned at a 4 µm thickness and stained with hematoxylin and eosin (H&E). The slides were reviewed by an experienced neuropathologist (C.G.E.) to assess for any evidence of local neurotoxicity or inflammation. Slides pertaining to two animals from each of the control (untreated), TMZ-loaded gel, resection + Empty gel, and resection + TMZ-loaded gel were reviewed. The reviewer was blinded to the groups.

### 2.10. Statistical Analysis

For the in vitro release, the experiments were performed in triplicates. For the in vivo efficacy studies, Kaplan–Meier curves were graphed using GraphPad Prism (version 9.5.0). The log-rank test was used for statistical analysis and was performed using IBM SPSS (version 29.0.2.0). A *p*-value cut-off of 0.05 was used for statistical significance.

## 3. Results

### 3.1. Polymer Characterization and Drug Release

An injectable polymeric matrix was developed based on a poly(anhydride-ester) system containing SA and RA at a 30:70 mass ratio, pSARA [13,14,15]. This formulation yields a soft, shear-thinning paste that can be readily administered by syringe. The selected monomer ratio was designed to modulate the relative contributions of ester and anhydride hydrolysis and degradation, resulting in controlled polymer surface erosion and sustained drug release. The polymer was synthesized via a one-pot condensation process, generating an alternating poly(SA-RA). Incorporation of APIs into the polymer matrix produced stable formulations with a uniform and homogeneous appearance. At high shear rates of 8.4–12.5 s^−1^, the formulation showed viscosities in the range of 8600–9000 cP, supporting consistent syringeability. Upon exposure to an aqueous physiological environment, pSARA systems are widely reported to undergo surface-dominated erosion, as described in previous studies on similar poly(anhydride-ester) materials [12,18,23,24]. The release kinetics are governed by the synchronization of polymer degradation and the properties of the loaded APIs [13,14,15].

**Figure 1:**
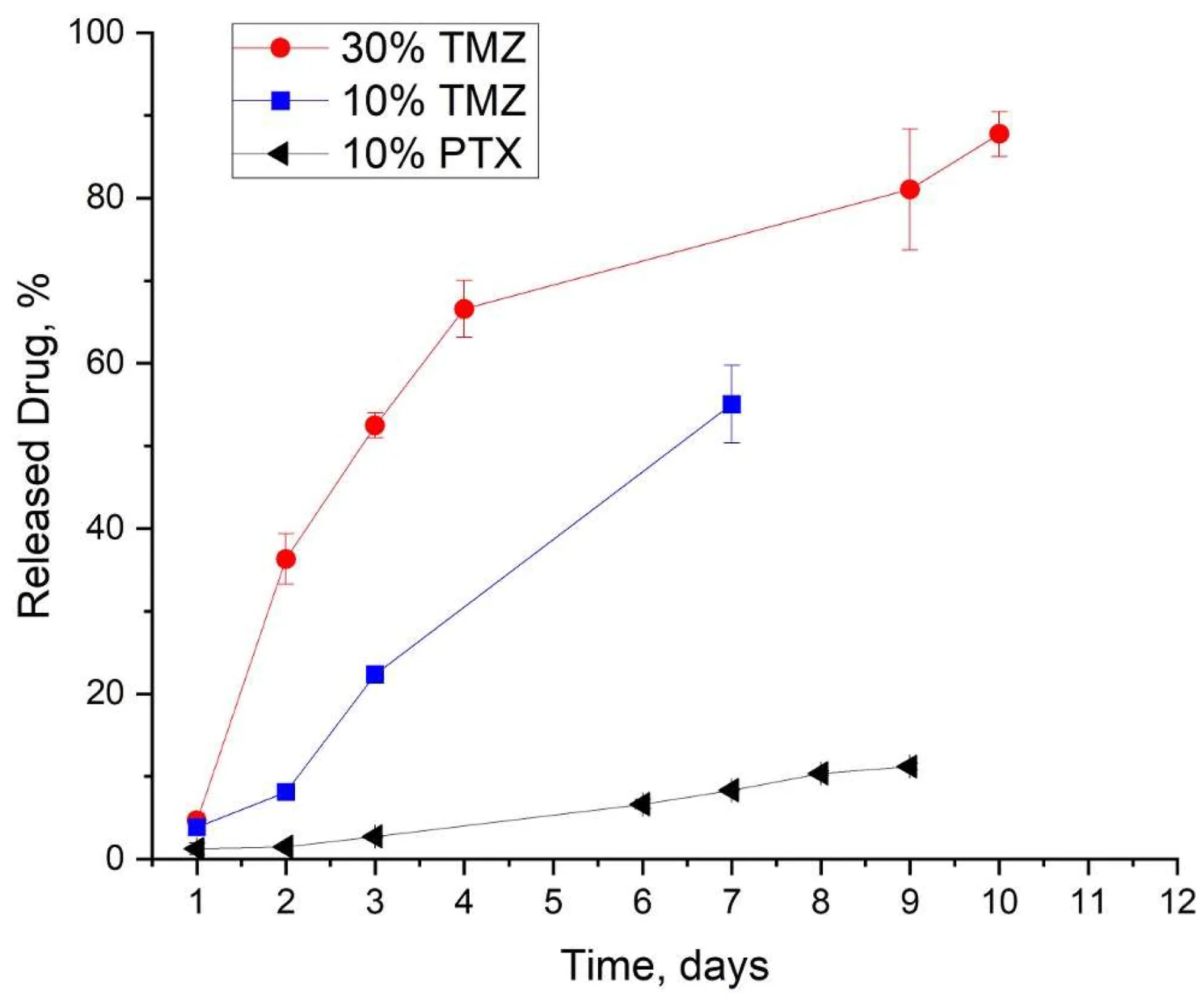
Gradual sustained release from the poly(sebacic acid–ricinoleic acid) poly(anhydride-ester) (pSARA) formulations. In vitro release profiles of 10% and 30% temozolomide (TMZ) formulations and a 10% paclitaxel (PTX) formulation. TMZ-loaded gels were evaluated in acetate buffer (pH 5) at 37 °C, while PTX-loaded gels were assessed in physiological phosphate-buffered saline (pH 7.4).

The in vitro release profiles of TMZ and PTX from their respective formulations are shown in Figure 1. The release of TMZ, a hydrophilic drug, is sensitive to drug loading concentrations. The 30% TMZ formulation exhibited a robust release profile, exceeding 55% cumulative release by day 3. In contrast, the 10% TMZ formulation demonstrated a more moderate profile, reaching approximately 20% over the same duration.

The relatively rapid release observed at higher TMZ loadings is attributed to the drug’s high solubility; as TMZ dissolves, it generates microvoids within the matrix [25], which facilitate water penetration and promote erosion of the polymer network through enhanced water ingress. For the hydrophobic drug PTX, the pSARA system provides only ~10% cumulative release over the initial 3 days in physiological buffer at a loading of 10%.

### 3.2. TMZ-Loaded pSARA Gel Shows Efficacy as a Monotherapy

**Figure 2:**
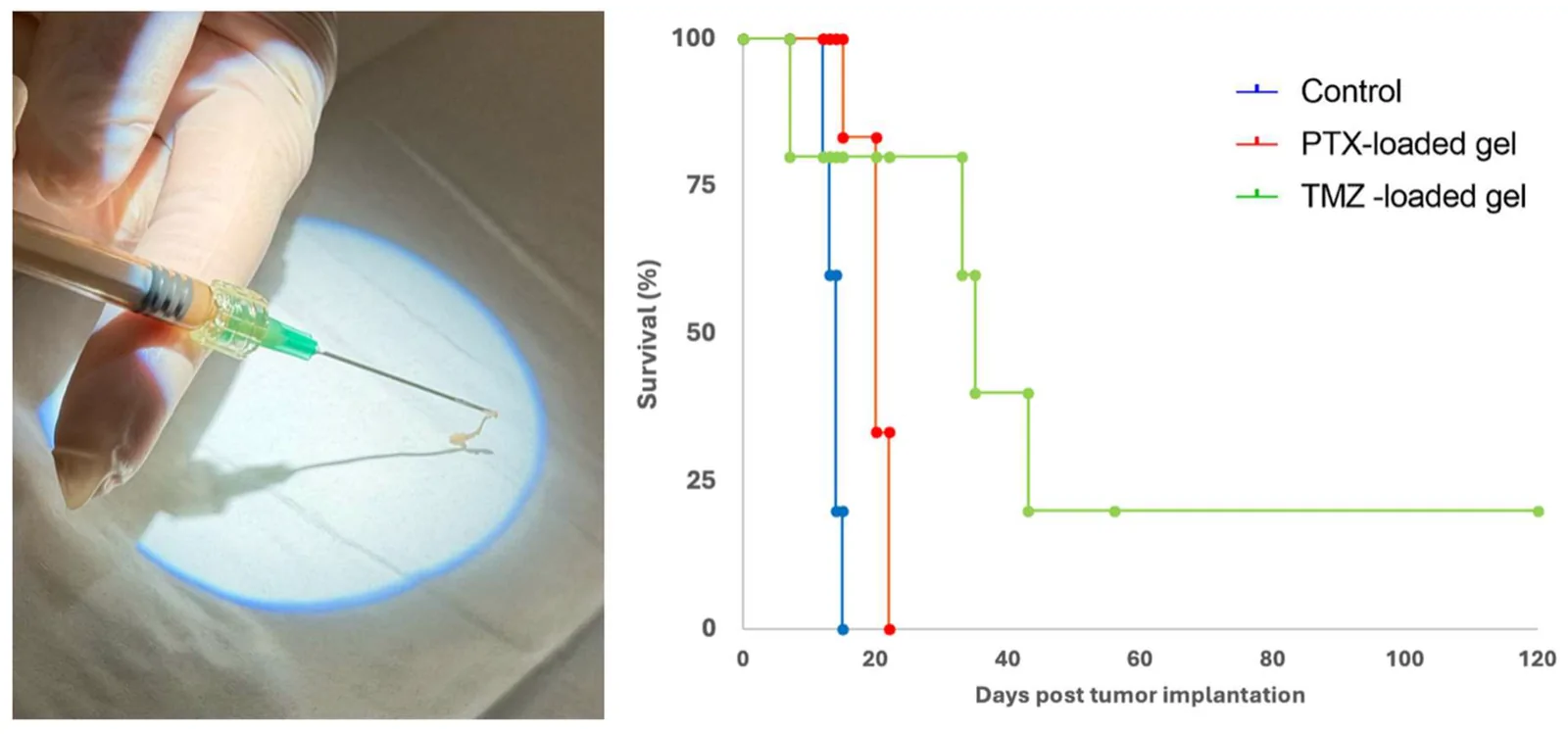
Syringeability and in vivo efficacy of the TMZ-loaded gel. **Left** panel: picture showing the syringeability of the TMZ-loaded gel at room temperature. **Right** panel: Kaplan–Meier curve showing the survival of rats implanted with 9L tumors and treated 5 days later with intratumoral injection of the TMZ-loaded or the PTX-loaded gel. Log-rank test *p*-value: TMZ-loaded gel vs. Control = 0.037; PTX-loaded gel vs. Control = 0.002; TMZ-loaded gel vs. PTX-loaded gel = 0.033. TMZ, temozolomide; PTX, paclitaxel.

We investigated the comparative efficacy of two pSARA-based therapeutic gels, one loaded with TMZ and one loaded with PTX in an orthotopic 9L gliosarcoma model. The rats were implanted with 9L tumor pieces intracranially and, five days later, treated with an intratumoral injection of either 30 µL of the TMZ-loaded or the PTX-loaded pSARA gel. Both therapies showed a significant prolongation in overall survival as compared to the control group (Figure 2), which received no intervention (log-rank test *p*-value for PTX-loaded gel vs. control = 0.002 while that for TMZ-loaded gel vs. control = 0.037). Nevertheless, the 30% TMZ-loaded gel showed a superior overall efficacy when compared to the PTX-loaded gel (log-rank test *p*-value = 0.033), with 20% of the rats receiving the former therapy achieving long-term survival (>120 days) as compared to none in the latter group. The median survival for the group receiving the TMZ-loaded gel was 35 days (95% confidence interval: 30.7–39.3 days), while that for group receiving the PTX-loaded gel was 20 days (95% confidence interval: 16.2–23.7 days), and for the control group was 14 days (95% confidence interval: 13.1–14.8 days). In a separate study, the 10% TMZ-loaded gel also resulted in a significant improvement in overall survival as compared to the control group (log-rank test *p*-value = 0.029); however, it was not as pronounced as that elicited by the 30% formulation (Supplementary Figure S1). Hence, the 30% TMZ-loaded formulation was selected for further testing and development.

### 3.3. TMZ-Loaded pSARA Gel Is Effective as an Adjuvant to Surgical Resection and Radiation Therapy

**Figure 3:**
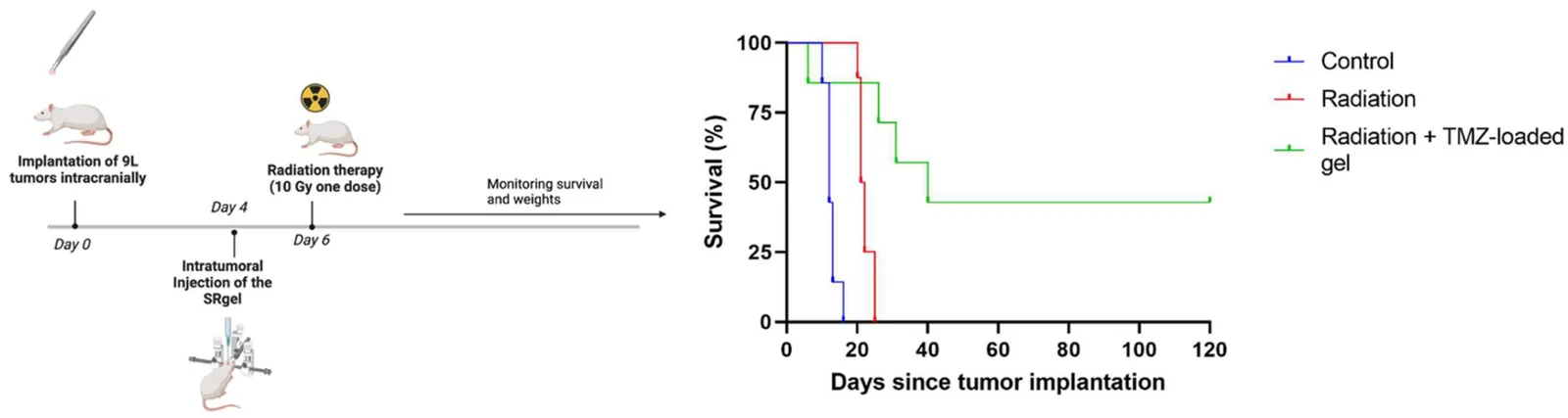
Efficacy of the TMZ-loaded gel in combination with radiation. **Left** panel: illustration showing the experimental procedure to test the combination of the TMZ-loaded gel with radiation therapy. Created in BioRender. Tyler, B. (2026) https://BioRender.com/u8wkc6u, accessed on 22 May 2026. **Right** panel: Kaplan–Meier curve showing the survival of rats implanted with 9L tumors and treated on day 4 with intratumoral injection of the TMZ-loaded and on day 6 with radiation therapy. Log-rank test *p*-value: Radiation vs. Control < 0.001; Radiation + TMZ-loaded gel vs. Control = 0.004; Radiation + TMZ-loaded gel vs. Radiation alone = 0.004. TMZ, temozolomide; SRgel, poly(sebacic-co-ricinoleic ester-anhydride) gel.

After the initial efficacy trial showing the superiority of the 30% TMZ-loaded gel as a monotherapy, we investigated its potential as a combination therapy with resection and radiation therapy, which are utilized in the clinical management of GBM. In specific, the combination of intratumoral injection of the TMZ-loaded gel with radiation therapy significantly improved the overall survival of rats implanted with the 9L tumors as compared to radiation therapy alone (log-rank *p*-value = 0.004) (Figure 3). The median survival for the untreated group was 12 days (95% confidence interval: 10.3–13.7 days), while that for the radiation group was 21 days (95% confidence interval: 19.9–22.1 days) and for the combination group was 40 days (95% confidence interval: 16.9–63.1 days). Moreover, Cox proportional hazards regression demonstrated that the addition of the TMZ-loaded gel to radiation therapy elicited a 91% reduction in the risk of mortality (hazards ratio (HR) = 0.089, *p*-value = 0.025). Additionally, the combination achieved a 42.8% long-term survival as compared to none in the radiation only group.

**Figure 4:**
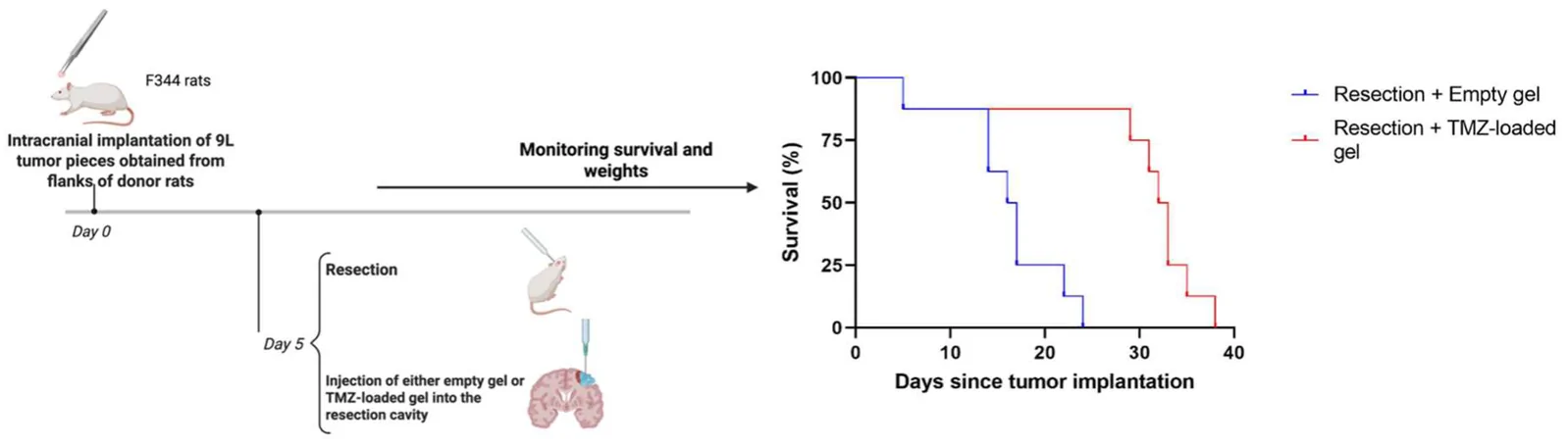
Efficacy of the TMZ-loaded gel in combination with surgical resection. **Left** panel: illustration showing the experimental procedure to test the combination of resection with either the TMZ-loaded gel or an empty gel. Created in BioRender. Tyler, B. (2026) https://BioRender.com/b3yl1ql, accessed on 22 May 2026. **Right** panel: Kaplan–Meier curve showing the survival of rats implanted with 9L tumors and treated on day 5 with surgical resection and intratumoral injection of either the TMZ-loaded gel or an empty gel. Log-rank test *p*-value: Resection + TMZ-loaded gel vs. Resection + empty gel < 0.001. TMZ, temozolomide.

In a similar manner, the combination of the TMZ-loaded gel with resection achieved a significantly increased survival as compared to resection combined with an empty gel (log-rank test *p*-value < 0.001) (Figure 4). The median survival for the group that received the empty gel after resection was 16 days (95% confidence interval: 13.2–18.7 days), while it was doubled (32 days; 95% confidence interval: 30.2–33.8 days) for the group that received the TMZ-loaded gel as an adjuvant. Herein, a Cox proportional hazards regression demonstrated that the addition of the TMZ-loaded gel to resection conferred a 94% reduction in the risk of mortality (HR = 0.063, *p*-value = 0.011).

### 3.4. Histological Assessment

The H&E-stained slides were assessed by a blinded neuropathologist. Spontaneous palisading necrosis was noted across the control, TMZ-loaded gel, resection + Empty gel, and resection + TMZ-loaded gel groups. Mild macrophage infiltration and hemosiderin deposition were noted in the TMZ-loaded gel and resection + TMZ-loaded gel groups. Nevertheless, no signs of neurotoxicity or excessive inflammation were noted in any of the slides.

## 4. Discussion

GBM remains among the most challenging solid tumors to treat, with a high likelihood of recurrence. The challenges are caused by several factors, including the presence of the BBB that precludes the delivery of a wide array of therapeutic agents. The advent of local delivery using biodegradable polymeric implants opened the door for ways to overcome the BBB and ensure the effective delivery of desired drugs. Since the FDA approval of carmustine wafers (Gliadel^®^), several local delivery formulations have been investigated to improve therapeutic drug delivery to brain tumors [26,27,28]; however, none have yet been added to clinical practice yet. Despite the preclinical efficacy of multiple products and technologies, different obstacles along the path towards first-in-human trials and subsequent clinical translation have halted their development. Hence, our focus was to test and optimize the pSARA delivery platform, which is already in clinical trials for head and neck cancers (NCT05200650), in order to facilitate the path towards its use to benefit patients with GBM.

TMZ has been a mainstay in the clinical management of GBM, and previous studies have established that the local delivery of this drug supersedes its oral administration in terms of efficacy [29,30]. PTX is a potent chemotherapeutic agent that has shown great efficacy in different types of cancer; however, it faces the issue of transport across the BBB [31]. Local delivery technologies that release PTX have demonstrated good efficacy [32,33], such as OncoGel, whose development was halted in phase I trials due to a “sponsor business decision, not based on safety or efficacy data” (NCT00479765). Hence, these two drugs were chosen as the leading candidates to test the pSARA delivery system in the context of GBM. And while both the TMZ-loaded and the PTX-loaded pSARA gels showed a significant prolongation of survival in the rat brain tumor model, the former showed greater efficacy and was chosen for further studies. The TMZ-loaded gel also demonstrated that it can be used in combination with surgical resection or radiation therapy to augment the efficacy of those modalities and further improve survival outcomes, with a 42.8% long-term survival achieved in the aggressive 9L rat tumor model when the combination of radiation therapy and the TMZ-loaded gel was used.

The local delivery of chemotherapeutic agents carries several advantages including increased efficacy and minimal risk for systemic toxicity and off-target effects. In specific, the commencement of oral TMZ therapy following surgical resection of GBM is usually delayed for 4–8 weeks (median 40 days) [34] to allow for recovery and proper wound healing from surgery. However, it has been observed that around half of the patients with GBM experience tumor regrowth during this interval, which is known as rapid early progression (REP) [35]. This can explain why early interventions, such as locally delivered chemotherapies, can be more effective than those requiring a delay before commencement [36]. Alternatively, local chemotherapy-releasing implants can act as a bridging therapy that prevents or slows down REP while patients recover and are preparing to begin systemic chemotherapy and radiation. Another advantage for locally delivered chemotherapies is their limited effect on systemic functions. Notably, previous studies have shown that the use of locally delivered chemotherapy can result in augmented immune activation and a greater efficacy for immune therapies as compared to systemic chemotherapy [37], which is known to exhaust the immune capacity. In fact, an ongoing clinical trial is investigating the combination of immune checkpoint inhibitors with carmustine wafers and radiation with or without oral TMZ (NCT05083754). Finally, a recent study has shown that the local delivery of TMZ can potentially overcome the resistance imparted by the expression of the *MGMT* gene, which is a known determinant for clinical outcomes with systemic TMZ [38].

Despite the promising findings that highlight the translational potential of the TMZ-loaded pSARA gel, the current manuscript has several limitations. In terms of in vitro release kinetics, the difference in release media used may limit the direct comparison of the release kinetics for the TMZ- and PTX-loaded formulations. Additionally, the release kinetics are not assessed in vivo, which should be tackled in future investigations involving higher animal models. This study shows efficacy in one rat model of GBM and lacks a direct comparison to systemic TMZ, hence, further comparative testing in other rodent and higher animal models is required to establish its translational potential. Finally, the mechanistic underpinnings that mediate the improvement in survival when the TMZ-loaded pSARA gel is combined with radiation have not been explored in this study and warrant further investigation in the future.

## 5. Conclusions

The findings discussed in this manuscript show the utility of the pSARA formulation as a local delivery mechanism in the context of GBM. Our investigations focused on the TMZ-loaded gel and have demonstrated its efficacy as a monotherapy and as an adjuvant to surgical resection and radiation. Given these encouraging results and the advantages of local delivery, it would be valuable to explore how the pSARA formulation can be further tested and eventually used to advance the management of GBM patients.

## Data Availability

Data is available upon request from the corresponding authors.

## Supplementary Materials

The following supporting information can be downloaded at: https://www.mdpi.com/article/10.3390/pharmaceutics18080963/s1, Figure S1. Kaplan-Meier curve showing the survival of rats implanted with 9L tumors and treated 5 days later with intratumoral injection of the 10% TMZ-loaded gel. Figure S2. TMZ chromatogram comparison for the TMZ API (as is) and the TMZ-loaded formulation after sterilization.

## Abbreviations

The following abbreviations are used in this manuscript:

BBB	Blood–brain barrier
FDA	Food and Drug Administration
GBM	Glioblastoma
HR	Hazards ratio
PBS	Phosphate-buffered saline
pSARA	poly(sebacic acid–ricinoleic acid) poly(anhydride-ester)
PTX	Paclitaxel
RA	Ricinoleic acid
REP	Rapid early progression
SA	Sebacic acid
SARRP	Small Animal Radiation Research Platform
TMZ	Temozolomide
