# Supplementary material for: Local Sustained Delivery of Temozolomide via an Injectable Poly(Anhydride-Ester) Depot for Glioblastoma Therapy
Source: Pharmaceutics. 2026 Aug 5;18(8):963. doi: 10.3390/pharmaceutics18080963 (PMC13516226; doi:10.3390/pharmaceutics18080963)
Supplement: Supplementary file 1 [file pharmaceutics-18-00963-s001.zip › pharmaceutics-4375475-supplementary.pdf]

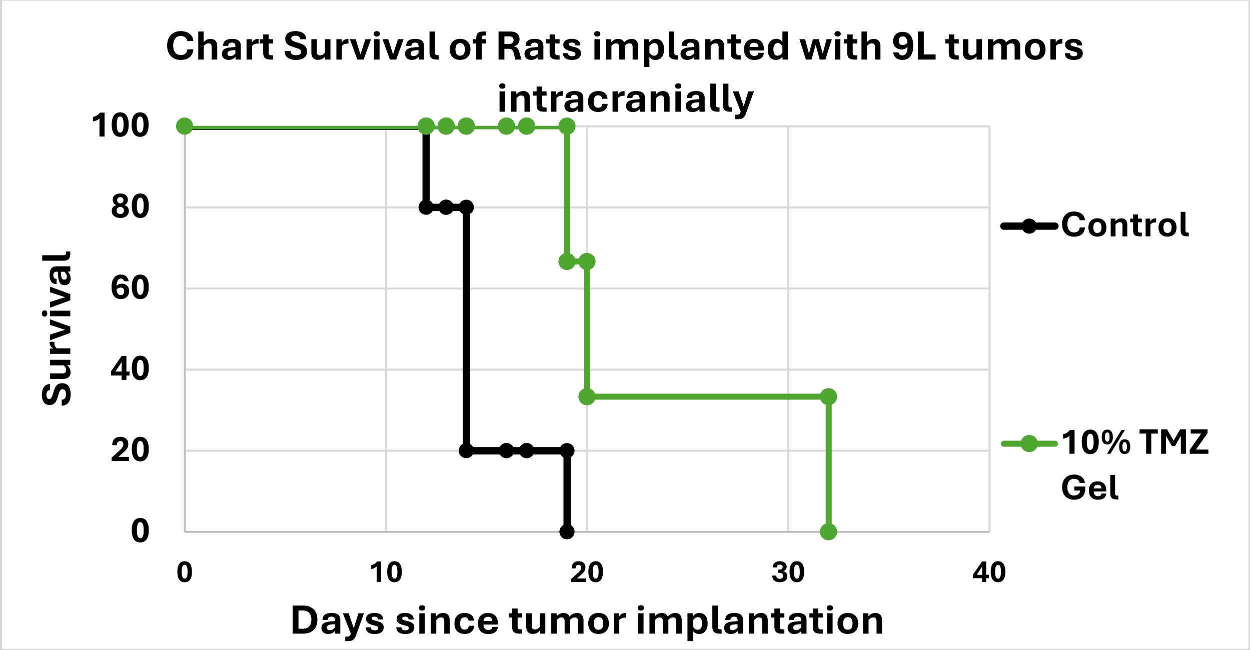

Supplementary Figure S1. Kaplan-Meier curve showing the survival of rats implanted with 9L tumors and treated 5 days later with intratumoral injection of the 10% TMZ-loaded gel. Log rank test p-value = 0.029.

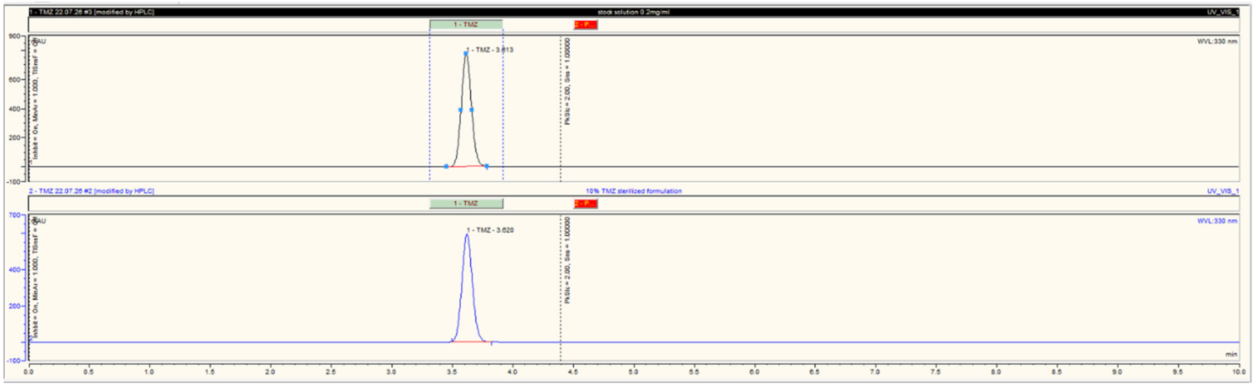

Supplementary Figure S2. TMZ chromatogram comparison for the TMZ API (as is) and the TMZ-loaded formulation after sterilization. As shown in the chromatograms, the retention time of TMZ is maintained and remains consistent between the API reference sample and the sterilized TMZ-loaded formulation. The absence of any shift in retention time can confirm that the chemical identity of TMZ is preserved following the sterilization process.
